# Hyperbaric oxygen protects type II collagen in interleukin-1β-induced mandibular condylar chondrocyte via inhibiting the JNK/c-Jun signaling pathway

**DOI:** 10.18632/oncotarget.19294

**Published:** 2017-07-17

**Authors:** Qi Sun, Gaoyi Wu, Hang Chen, Lei Chen, Hongyu Chen, Guoxiong Zhu, Huaqiang Zhao

**Affiliations:** ^1^ School of Stomatology, Shandong University, Jinan City, Shandong Province, China; ^2^ Shandong Provincial Key Laboratory of Oral Tissue Regeneration, Jinan City, Shandong Province, China; ^3^ Department of Stomatology, Jinan Military General Hospital, Jinan City, Shandong Province, China; ^4^ Present address: Department of Oral and Maxillofacial Surgery, School of Stomatology, Shandong University, Jinan City, Shandong Province, China

**Keywords:** hyperbaric oxygen, IL-1β, JNK, COL2, Sox-9

## Abstract

The aim of this study was to explore the mechanisms of Hyperbaric oxygen (HBO) protective on interleukin-1β (IL-1β) induced rat's mandibular condylar chondrocytes. Chondrocytes were exposure to Hyperbaric oxygen after induced inflammatory by IL-1β. After that, the expression of p-JNK and c-Jun was increased significantly, while the Sox-9 was decreased significantly, Immunofluorescence results showed that the expression of p-JNK and p-c-Jun were decreased while the expression of Sox-9 and COL2 were increased in chondrocytes treated with IL-1β and selective JNK inhibitor. Hyperbaric oxygen might plays similar roles with the JNK-specific inhibitor SP600125, inducing the increase of Sox-9 and COL2 expression. On the whole, IL-1β induced inflammatory in chondrocytes by activating the JNK/c-Jun signaling pathway and down-regulate the expression of Sox-9 and COL2. However, Hyperbaric oxygen can inhibits IL-1β induced inflammatory response in chondrocytes though block the JNK/c-Jun signaling pathway and up-regulate the expression of Sox-9 and COL2.

## INTRODUCTION

OA is the commonest joint disease that characterized by the progressive degeneration of articular cartilage and finally the destruction of joint [1]. Temporomandibular joint (TMJ), the most important joint of maxillofacial, is susceptible to OA. Vascular tissue was absent in normal articular cartilage in the hypoxia environment [2]. The oxygen content of normal cartilage is higher than which of the OA cartilage [3, 4]. The degradation of the extracellular matrix (ECM) is a critical process in the development of osteoarthritis [5]. Temporomandibular joint osteoarthritis (TMJOA) is a degenerative joint disease characterized by the death of chondrocytes, the loss of cartilage ECM, and the subchondral bone resorption in its early stages, followed by abnormal reparative bone turnover [6]. However, the mechanism of TMJOA remains unclear. A critical source of cartilage is the differentiation from mesenchymal cells to chondrocytes, which serves as a template for long bone formation [7]. Several studies have reported that TMJOA could occur the erosion of articular cartilage which predominantly resulting the imbalance between chondrocyte-controlled catabolic and anabolic processes [8, 9].

In the patients with OA, interleukin-1β (IL-1β) and tumor necrosis factor α (TNF-α) were highly expressed in articular fluid, it was considered as the most important pro-inflammatory cytokines in the process of OA [10]. It was reported that neutralize IL-1β could prevent the destruction of cartilage and bone, but neutralizing TNF-α could only reduce the synovial inflammation, useless for the cartilage damage [11]. Gene therapy studies demonstrated that IL-1 receptor antagonist (IL-1Ra) could significantly relieve the synovial inflammation and inhibit the degradation of cartilage matrix [12]. Human studies confirmed that the levels of IL-1βsignificantly increased in the synovial fluid of OA patients, which suppressed the synthesis of the major ECM proteins COL2 and Aggrecan (AGG) [13]. Therefore, IL-1β is generally considered as an agent that induces chondrocyte inflammatory state [14–16].

JNK, a major group of the mitogen-activated protein kinase (MAPK) family, acts as molecular targets for anti-inflammatory therapy [17]. Phosphorylation of JNK activates inflammatory factors genes expressions, such as TNF-α and nitric oxide (NO) [18]. A recent study suggested that the JNK signaling pathway was crucial in cell differentiation, apoptosis, and proliferation as well as joint injury activated by interleukin-1 (IL-1), TNF-α, and other inflammatory factors [19]. c-Jun, a member of the activator protein-1 (AP-1) transcriptional complex, is preferentially phosphorylated and activated by JNK and is central in the up-regulation of several matrix metalloproteinases (MMPs) that promote the destruction of OA chondrocytes [20]. Sox-9 is the transcription factor of SRY gene family, which plays an important role in the growth and development of cartilage, and it is considered as the main control factor in cartilage formation. Embryonic stem cells with homozygous defect in Sox-9 do not differentiate into cartilage cells [21]. COL2 and AGG, specific transcription factors of cartilage, can be activated by the complex of Sox-9 with L-sox-5 and sox-6, and differentiate mesenchymal stem cells into chondrocytes [22]. It was reported that suppression of Sox-9 expression contributes to the inhibition of COL2 expression [23].

HBO treatment leads to new vasoconstriction and hyper oxygenation, making it as an effective treatment for various clinical disorders [24]. Previous reports suggested that HBO treatment decreases the IL-1βsecretion from monocytes [25], suppresses the apoptosis of chondrocytes and pro-inflammatory cytokines [26]. The results suggested that HBO treatment increased the AGG and COL2 expression [26]. However, the effects and potential molecular mechanism of HBO on IL-1β-induced mandibular condylar chondrocytes in TMJOA remains unclear.

We explored whether HBO could suppress the inflammation process and increase the secretion of COL2 in IL-1β-induced rat mandibular condylar chondrocytes. The underlying molecular mechanism of JNK/c-Jun signaling pathway in HBO protective of IL-1β-induced rat mandibular condylar chondrocytes was investigated.

## RESULTS

### Identification of normal mandibular condylar chondrocytes

**Figure 1:**
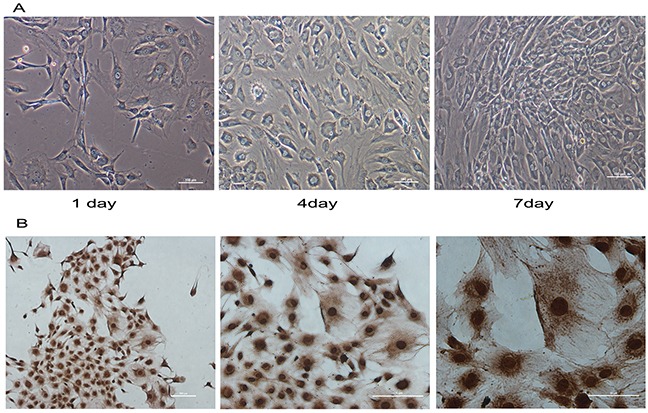
Immunohistochemistry and morphology identify of mandibular condylar chondrocytes Normal condylar chondrocytes special expressed COL2. **(A)** The morphology of normal chondrocytes cultured for 1, 4and 7 days was observed under a microscope. **(B)** The immunohistochemical staining shown that COL2 was positive in the chondrocytes.

Normal chondrocytes were circular, polygonal and star shaped, and the cell nuclei located in the central of abundant cytoplasm (Figure 1A). Immunohistochemical staining for COL2 was positive in the chondrocytes (Figure 1B). Thus, the cells were identified as chondrocytes.

### IL-1β induces JNK/c-Jun activation and inhibits Sox-9 and COL2 expressionin mandibularcondylar chondrocytes

**Figure 2:**
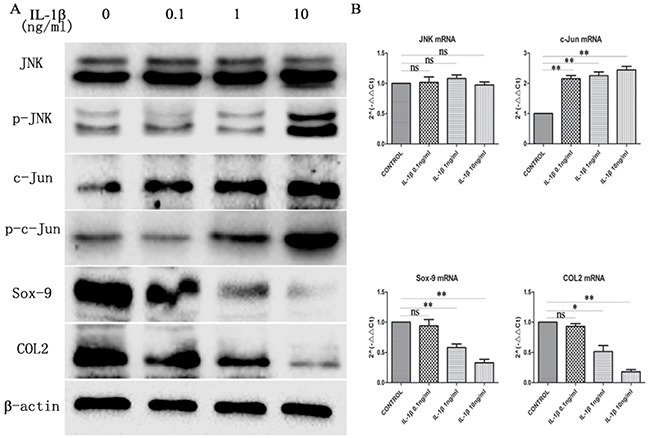
The expression of JNK, p-JNK, c-Jun, p-c-Jun, Sox-9 and COL2 in chondrocytes treated with different concentration of IL-1β **(A)** Western blot was used to JNK, p-JNK, c-Jun, p-c-Jun, Sox-9 and COL2 protein levels in chondrocytes treated with 0, 0.1, 1 and 10ng/ml IL-1β. Bands were quantified using Quantity One 5.0. **(B)** The mRNA levels of JNK, c-Jun, Sox-9 and COL2 in the chondrocytes. The 2^−ΔΔCt^ method was adopted with GAPDH as the reference gene. Bars represent the mean ± SD of each group (n = 6). As a control, the membrane was stripped and incubated with β-actin. Significant differences between the groups are marked with asterisks (*P< 0.05, **P< 0.01); ns: no statistical significance.

IL-1β, a major inflammatory cytokine, destroys cartilage in chondrocyte inflammatory responses. To investigate the relationship between the JNK/c-Jun signaling pathway and the concentration of IL-1β, three concentrations of IL-1β (0.1ng/ml, 1ng/ml, and 10ng/ml) were selected to stimulate mandibular condylar chondrocytes for 24h. IL-1β potently activated the JNK/c-Jun signaling and inhibited Sox-9 and COL2 expression with IL-1β concentration-dependent manner in mandibular condylar chondrocytes. The western blot analysis revealed that the expression of p-JNK and p-c-Jun was significantly increased while the expression of COL2 and Sox-9 was obviously decreased in the IL-1β treated groups in a dose dependent manner. The JNK expression was no obviously changed between all groups. The mRNA expression of c-Jun was increased significantly in the IL-1β groups compared with the control group. However, no significant difference between the three IL-1β groups (Figure 2A). RT-qPCR analysis also revealed that the mRNA levels of COL2 and Sox-9 in the 1ng/ml IL-1β group and 1ng/ml IL-1β group were significantly lower than that of control group (*P* < 0.01). The expression of c-Jun (*P* < 0.01) was significantly elevated in the three IL-1β groups compared with the control group (Figure 2B). These results showed thatIL-1β induced the activation of JNK/c-Jun and inhibited Sox-9 and COL2 expression in mandibular condylar chondrocytes in a concentration dependent manner.

### IL-1β induced inhibit the expression of Sox-9 and COL2 were actived by the JNK/c-Jun signaling pathway

**Figure 3:**
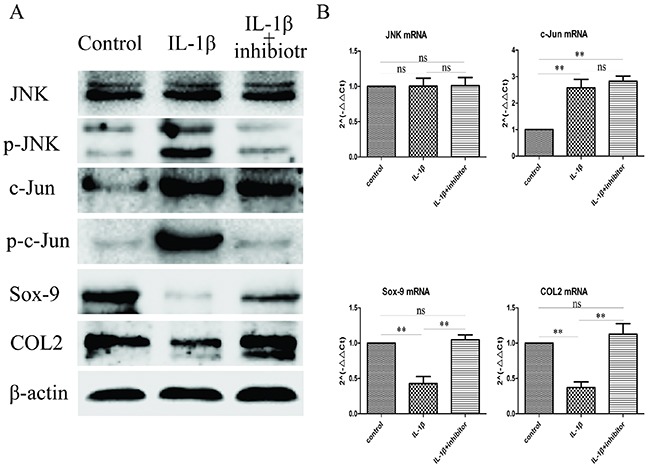
Inhibit JNK/c-Jun signaling pathway increase Sox-9 and COL2 expression **(A)** Western blot was used to evaluate the change of JNK, p-JNK, c-Jun, p-c-Jun, Sox-9 and COL2 protein levels in different groups. **(B)** Relative levels JNK, c-Jun, Sox-9 and COL2mRNA in the chondrocytes. The 2^−ΔΔCt^ method was adopted with GAPDH as the reference gene. Bars represent the mean ± SD of each group (n = 6). As a control, the membrane was stripped and incubated with β-actin. Significant differences between the groups are marked with asterisks (*P< 0.05, **P< 0.01); ns: no statistical significance.

Previously, we generated the JNK/c-Jun and Sox-9 and COL2 expression profile in mandibular condylar chondrocytes by using western blot and RT-qPCR, and the expression of JNK/c-Jun andSox-9 and COL2 expression have a striking resemblance. These findings indicate that Sox-9 and COL2 expression might associated with JNK/c-Jun signaling pathway. To investigate whether Sox-9 and COL2 expression was dependent on the activation of JNK/c-Jun in mandibular condylar chondrocytes, we treated the mandibular condylar chondrocyte with the JNK inhibitor SP600125 to suppressed the expression of Sox-9 and COL2 induced by IL-1β (10 ng/ml). The result of western blot analysis revealed that the protein expression of Sox-9 and COL2 were significantly increased and the expression of p-JNK and p-c-Jun were significantly decreased in the IL-1β+inhibitor group compared with the IL-1βgroup, and the protein expression of JNK and c-Jun were no obviously changes (Figure 3A). The result of RT-qPCR analysis revealed that the mRNA expression of Sox-9 (*P*< 0.01) and COL2(*P*< 0.05) were significantly increased and the mRNA expression of JNK and c-Jun were no statistical significance in the IL-1β+inhibitor group compared with the IL-1β group (Figure 3B). These results indicate that the Sox-9 and COL2 expression was dependent on the activation of JNK/c-Jun in IL-1β-induced mandibular condylar chondrocytes.

### HBO protected the expression of Sox-9 and COL2 of IL-1β-induced chondrocytes

**Figure 4:**
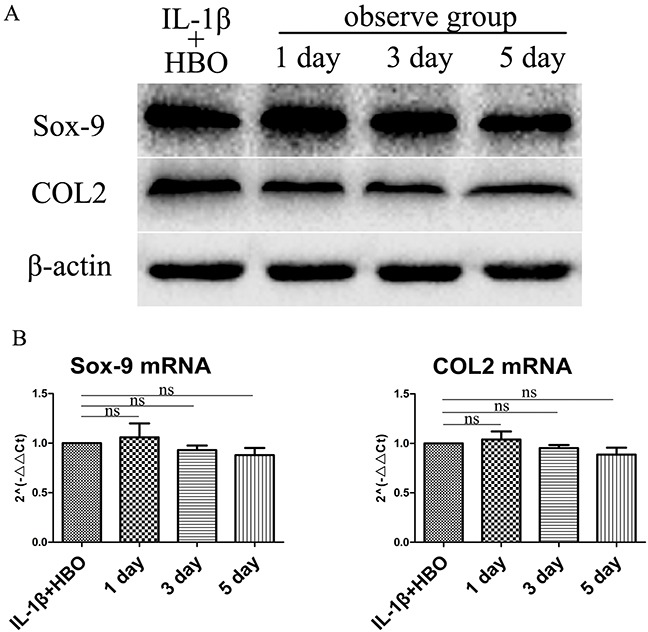
Western blot and RT-qRCR showed the change in expression of Sox-9 and COL2 with HBO treatment **(A)** Western blot was used to evaluate the change in expression of Sox-9 and COL2 protein levels in different groups. **(B)** Relative levels Sox-9 and COL2mRNA in the chondrocytes. The 2^−ΔΔCt^ method was adopted with GAPDH as the reference gene. Bars represent the mean ± SD of each group (n = 6). As a control, the membrane was stripped and incubated with β-actin. Significant differences between the groups are marked with asterisks (*P< 0.05, **P< 0.01); ns: no statistical significance.

The effect of HBO on Sox-9 and COL2 of IL-1β-induced chondrocytes were investigated with western blotting and RT-qPCR. The results (Figure 4A, 4B) revealed that the proteins expression and mRNA expression of Sox-9 and COL2were not significantly changed compared with the HBO group.

### HBO increased the expression of Sox-9 and COL2 of IL-1β-induced chondrocytes via inhibit the JNK/c-Jun signaling pathway

**Figure 5:**
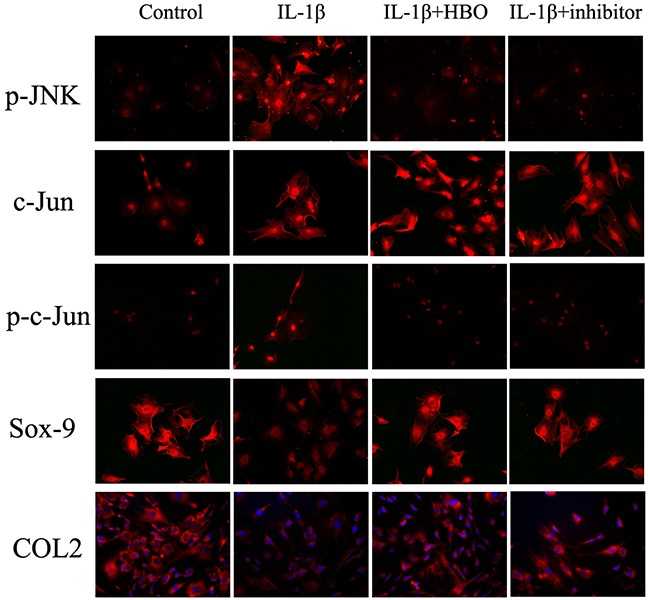
Immunofluorescence showed the change in expression of p-JNK, c-Jun, p-c-Jun, Sox-9 and COL2 Immunofluorescencewith six replicates showing the change in expression of p-JNK, c-Jun, p-c-Jun, Sox-9 and COL2 in the different groups.

**Figure 6:**
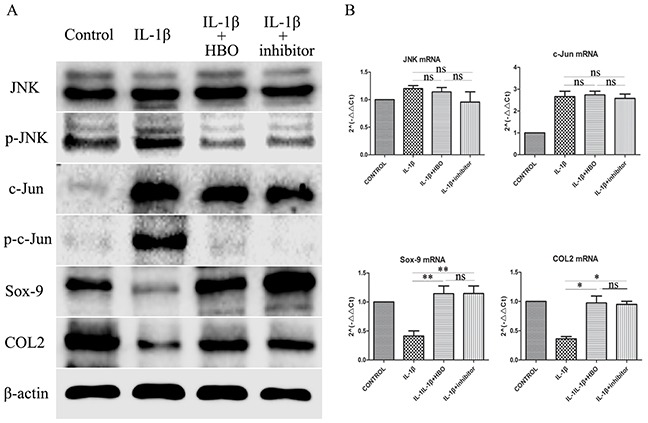
Western blot and RT-qRCR showed the change in expression of JNK/c-Jun signaling pathway, Sox-9 and COL2 **(A)** Western blot was used to evaluate the change in expression of JNK, p-JNK, c-Jun, p-c-Jun, Sox-9 and COL2 protein levels in different groups. **(B)** Relative levels JNK, c-Jun, Sox-9 and COL2mRNA in the chondrocytes. The 2^−ΔΔCt^ method was adopted with GAPDH as the reference gene. Bars represent the mean ± SD of each group (n = 6). As a control, the membrane was stripped and incubated with β-actin. Significant differences between the groups are marked with asterisks (*P< 0.05, **P< 0.01); ns: no statistical significance.

To investigate whether HBO increased the expression of Sox-9 and COL2 of IL-1β-induced chondrocytes via inhibit the JNK/c-Jun signaling pathway. The levels of expression of JNK, p-JNK, c-Jun, p-c-Jun, Sox-9 and COL2 were measured by immunofluorescence, western blot and RT-qPCR. It revealed that the positive expression areas of p-JNK (*P*< 0.05), c-Jun (*P*< 0.05) and p-c-Jun (*P*< 0.05) were significantly increased and the positive expression areas of Sox-9 (*P*< 0.05) and COL2 (*P*< 0.05) were significantly lower in the IL-1β group compared with the control group (Table 1). In the HBO group, the IOD and positive expression areas of Sox-9 andCOL2 were significantly higher and the positive expression areas of p-JNK (*P*< 0.05), c-Jun (*P*< 0.05) and p-c-Jun (*P*< 0.05) were significantly lower than that of the IL-1β group (Figure 5). To confirm the expression quantity in the chondrocytes, the above proteins were quantified by western blot analysis (Figure 6A). The result of western blot was complied with the result of immunofluorescence. The result of RT-qPCR analysis revealed that the mRNA expression of Sox-9 (*P*< 0.01) and COL2 (*P*< 0.05) were significantly higher and the mRNA expression of JNK and c-Jun were no statistical significance in the HBO group compared with the IL-1β group (Figure 6A, 6B).

**Table 1:** Primers used for RT-qPCR

Primer name	Sequence
JNK(forward)	5′-CTCTCCAGCACCCGTACATC-3′
JNK(reverse)	5′-CGCCATTCTTAGTTCGCTCC-3′
c-Jun(forward)	5′-GCACATCACCACTACACCGA-3′
c-Jun(reverse)	5′-TATGCAGTTCAGCTAGGGCG-3′
Sox-9(forward)	5′-CTCCTACTACAGCCACGCAG-3′
Sox-9(reverse)	5′-AGCTGTGTGTAGACGGGTTG-3′
COL2(forward)	5′-AGGAGCAAGGAGAAGAAG -3′
COL2(reverse)	5′-TTACAGTGGTAGGTGATG-3′
GAPDH(forward)	5′-ATGATTCTACCCACGGCAAG-3′
GAPDH(reverse)	5′-CTGGAAGATGGTGATGGGTT-3′

## DISCUSSION

As the most significant cell of mandibular condylar cartilage, mandibular condylar chondrocyte plays an important role in the formation of ECM. The ECM degradation is crucial in the development of OA. HBO, as an efficient therapy, widely used in clinical medicine, has potential use for regeneration and heal therapy. Nevertheless, the mechanism of HBO in most clinical therapy remains unclear. Previous studies had shown that HBO treatment could suppress the secretion of IL-1βfrom monocytes and inhibits the apoptosis in osteoarthritic chondrocytes [25, 26]. However, there is no report about the effects of HBO in TMJOA. Our study demonstrated that HBO treatment inhibited the inflammation process in the IL-1β-induced rat mandibular condylar chondrocytes through inhibition of JNK/c-Jun signaling pathway and increased the expression of Sox-9 and COL2.

IL-1β, an inflammatory cytokine, induces several mediators of cartilage degradation and plays an important role in the pathogenesis of OA [27, 28]. Therefore, IL-1β was commonly use for induced chondrocytes inflammatory in several research [29, 30]. Normal chondrocyte's ECM contains a large amount of COL2, which is typically used as a mark for the identification of chondrocytes [5]. The degradation of COL2 is as a typically reflect the cartilage degradation in OA [31]. Our study provided the evidence that HBO inhibits the inflammatory process via inhibit IL-1β induced down expression of COL2 in chondrocytes and it may play a protective role against IL-1βrelevant incidence and development of TMJOA. The JNK/c-Jun signaling pathway has been as a major regulator in inflammation response [32]. It was reported that inhibiting the expression of JNK/c-Jun signaling pathway effectively reduced NO-induced injury [33, 34].

Previous researches have shown that the JNK/c-Jun signaling pathway was activating by inflammatory factors and the inhibition of it could suppress the cartilage degradation [35, 36]. The activation of JNK/c-Jun signaling pathway results in the expression of MMPs and then breaks chondrocytes of cartilage [37]. In the present study, the phosphorylation of JNK was increased with the increasing IL-1β concentration. We inferred that it is the effect of stress reaction and auto regulation with the low expression of p-JNK in low IL-1β concentration. In addition, Sox-9, one of the factors up-regulation COL2, was affected by IL-1β and inhibited the JNK/c-Jun signaling pathway with JNK-specific inhibitor SP600126 induced the expression of Sox-9 increased significantly.

Our data also showed that HBO played similar roles with the JNK-specific inhibitor SP600125, which leading to the expression of Sox-9 and COL2 in the IL-1β-induced mandibular condylarchondrocytesrats. We suggested that the effects of HBO might contain two mainly aspects that HBO can change the hypoxia environment of mandibular condylar chondrocytes and inhibition of inflammatory cytokines and mechanical stimulus. The oxygen content of normal cartilage was higher than that of the OA cartilage [3, 4]. Oxygen deficit can also cause inflammation factors release and strengthen the effect of polymorph nuclear leukocytes cytotoxic [38]. Mechanical stimulus has been confirmed not only to promote articular cartilage synthesis but also to inhibit catabolism [39, 40]. The pressure stimulation is passes through the receptors of cell surface. Integrins, a heterodimer mainly consisting of α and β subunit, are a family of cell adhesion molecules. Integrin acts as the mechanical stimulation for the surface receptors in cartilage cells which regulate proliferation, differentiation, stretching and migration of chondrocyte [41, 42]. Focal adhesion kinase (FAK), a cytoplasmic protein tyrosine kinase, can be activated by mechanical signals transmitted through the integrins [43]. The JNK/c-Jun signaling pathway is one of targets of FAK [44]. Although, we analyzed that the effect of HBO on the JNK signaling pathway but this still needs further research.

In summary, our study demonstrated that HBO treatment inhibits IL-1β-induced the inflammation response in rat mandibular condylar chondrocytes and promotes COL2 synthesis via inhibiting JNK/c-Jun signaling pathway. These finding suggested that HBO had a potential therapeutic function for treating TMJOA.

## MATERIALS AND METHODS

### Animals

All experimental procedures were carried out in accordance with the regulations and institutional guidelines of the Ethics Committee at the Stomatological Hospital of Shandong University (Permit Number: GD201509). All experimental protocols were approved by the Ethics Committee at the Stomatological Hospital of Shandong University Animals were performed under anesthesia, and all efforts were made to minimize the animal suffering. All the animals were sacrificed by an over dose of pentobarbital sodium after the experiment.

### Isolation and culture of rat TMJ chondrocytes

Chondrocytes were isolated from TMJ condylar cartilage from 3weeks old Wistar rats (Shandong University Animal Centre, Jinan, Shandong, China). The tissues was washed twice with phosphate-buffered saline (PBS), digested with 0.25% trypsin for 15 min, and digestion with 0.1% collagenase II in Dulbecco's Modification of Eagles Medium (DMEM) supplemented with20% FBS, 100 mg/ml penicillin, and 100 mg/ml streptomycin for 4h. Following incubation at 37°C in a humidified atmosphere of 5% CO_2_, the chondrocytes were collected at 2h intervals by centrifugation. Then, chondrocytes were resuspended in DMEM containing 20% fetal bovine serum (FBS) and supplemented with 1% penicillin and streptomycin. Cells were plated in 60 mm culture dish at a 1.0×10^6^cells/plate density. The medium was changed every 3 days. The second passage (P2) cells were harvested after primary culture for 7 days. Observe the morphology of chondrocytes under a microscope. Cells were treated with 0.1ng/ml, 1ng/ml and 10ng/ml recombinant rat IL-1β (Rocky Hill, NJ, USA) for 24h. To eliminate the influence of the serum IL-1β in the culture medium, it was replaced with serum-free culture medium cultured for 24 h before HBO treatment. Firstly, the cells were divided into four groups: control group with no treatment, other three IL-1β group were treated with 0.1ng/ml,1ng/ml,10ng/ml respectively. Secondly, the cells were divided into three groups: control group with no treatment, IL-1β treatment group (cells were stimulated with 10 ng/ml IL-1β for 24h); IL-1β+inhibitior group (cells were treated with 10 ng/ml IL-1β for 24h and then treated with 25μM SP600125 for 40min). Thirdly, the cells were divided into four groups: HBO treatment group (cells were stimulated with 10 ng/ml IL-1β for 24h and treated with HBO), observe groups (cells were stimulated with 10 ng/ml IL-1β for 24h and treated with HBO, after that change culture medium cultured for 1day, 3day, 5day in incubator with 5% CO_2_ /95% air with no HBO). Fourthly, the cells were divided into four groups: control group with no treatment, IL-1β treatment group (cells were stimulated with 10 ng/ml IL-1β for 24h), HBO treatment group (cells were stimulated with 10 ng/ml IL-1β for 24h and treated with HBO); IL-1β+inhibitior group (cells were treated with 10 ng/ml IL-1β for 24h and then treated with 25μM SP600125 for 40min).

### Immunohistochemistry

Chondrocytes were cultured in fibronectincoated chamber slides for 24h. The cells were fixed for 10 min in cold PBS containing 0.2M sucrose and 4% paraformaldehyde and washed twice with PBS. Added two drops of 3% H_2_O_2_-methanol to the slide and incubated at room temperature for 10 min and then washed with PBS for three times. Added goat serum (50-100μl) to the slide, then incubated at room temperature for 20 min. Added type II collagen antibody (50-100μl of a 1:200dilution) to the slide, then incubated at 37°C for 2h and washed three times with PBS before the addition of 50μ lintensifier. Then the slides were incubated at room temperature for 30min and washed three times with PBS. Added a universal immunoglobin G (IgG) antibody-Fab segment-horseradish peroxideasepolymer (50μl) to the slide, and then incubated at 37°C for 30 min and washed three times with PBS. Use 3,3′-diaminobenzidine solution for color development. The slides were washed for 15 min with tap water and then washed once with distilled water. Hematoxylin staining was performed according to a standard protocol.

### Cell treatment and exposure to HBO

Cells of control group and IL-1β group were cultured in incubator with 5% CO_2_/95% air with no HBO. Cells of HBO group were treated with IL-1β (10 ng/ml) for 24h and then incubated in a hyperbaric chamber (Billups-Rothenberg, Del Mar, USA) at 1.5 atmospheres absolute (ATA), and exposed to 100% O_2_ for 25min followed by room air for 5min with the total treatment time was 90 min per 48h. Cells of observation group were treated with IL-1β (10 ng/ml) for 24h and then incubated in a hyperbaric chamber 1.5 ATA, and exposed to 100% O_2_ for 25min followed by room air for 5min with the total treatment time was 90min per 48h, after that observe groups were changed normal culture medium cultured 1 day, 3 day, 5 day in incubator with 5% CO_2_ /95% air with no HBO.

### Immunofluorescence

Chondrocytes were cultured in 24 well slides for 24 h. Chondrocytes were washed three times with PBS and then were fixed with 4% paraformaldehyde in PBS for 20min at room temperature. Cells were blocked in PBS containing 0.1% Triton X-100 and 1% normal goat serum for 1h. Fixed cells were washed with PBS and incubated at 4°C overnight with the following primary antibodies: rabbit polyclonal type II collagen (1:1000 dilution, lot no. ab34712, Abcam), rabbit monoclonal c-Jun (1:200 dilution, lot no.9165, Cell Signaling Technology), rabbit monoclonal phosphor-c-Jun serine 73 (1:200 dilution, lot no.3270, Cell Signaling Technology), and rabbit polyclonal Sox-9 (1:200 dilution, lot no.HPA001758, Sigma). The cells were washed three times with PBS and incubated with the appropriate cy-3 secondary antibodies for 1h at room temperature. Then slides were washed three times with PBS, and cell nuclei were identified with Hoechst staining for 3min. Slides were washed a final three times in PBS and then observed with a Nikon photomicroscope.

### Western blot analysis

To isolate total chondrocytes protein, cells were washed with pre-chilled PBS and lysed with ice-cold lysis buffer containing: 50 mmol/l Tris-HCl, pH 7.4; 1% NP-40; 150 mmol/l NaCl; 0.1% sodium dodecyl sulfate (SDS); supplemented with proteinase inhibitors phenyl methanesulfonyl fluoride. The concentration of protein in the chondrocyte lysates was quantified with the BCA protein assay kit, and diluted to an equal concentration with loading buffer. A total of 40μg of protein was fractionated by 6%–10% sodium dodecyl sulfate-poly-acrylamide gel electrophoresis (SDS-PAGE) and then electrically blotted on to polyvinylidene fluoride (PVDF) membranes (Millipore, Billerica, MA, USA). The membranes were blocked with Tris-buffered saline (TBST) containing 5% skim milk for 1 h and hybridized with primary antibody against JNK (1:1000 dilution, Cell Signaling Technology), phosphor-JNK (1:1000dilution, Cell Signaling Technology), c-Jun (1:1000dilution, Cell Signaling Technology), phosphor-c-Jun (1:1000 dilution, Cell Signaling Technology), Sox-9 (1:500dilution, Sigma), COL2 (1:1000dilution, Abcam) and β-actin (1:1000dilution, Beyotime Biotechnology) overnight at 4°C. After washed 3 times with TBST, the membranes were hybridized with horseradish peroxidase (HRP)-conjugated anti-mouse or anti-rabbit secondary antibody (Bioworld Technology) for 2 h at room temperature. After washing three times again with TBST, protein was detected by enhanced chemiluminescence reagent (Beyotime Biotechnology). The optical density of the immunoblots was quantified using Quantity One software (Bio-Rad Laboratories).

### Reverse transcription and real-time quantitative polymerase chain reaction (RT-qPCR) analysis

Total RNA (5μg) was extracted from chondrocytes by using TRIzol reagent (Invitrogen) according to the manufacturer's instructions. First strand cDNA was synthesized by reverse transcription using Prime Script RT Reagent Kit (Takara Biotechnology) and real-time PCR was performed using SYBR Premix Ex Taq II (Takara Biotechnology) according to the manufacturer's instructions. RT-qPCR was carried out in 7500 Real Time PCR system (Applied Biosystems, USA) with the following settings: 10min of pre-incubation at 95°C followed by 40 cycles of 20s at 95°C and 60 s at 55°C. Primers were designed with Primer Premier Version 5.0 software and their efficiency was confirmed by sequencing their conventional PCR products. The primer sequences were showed in Table 2. Melting curve analysis was carried out using the default program. After each reaction, the cycle threshold (Ct) was recorded when the amplification curve reflected the exponential kinetic measurements. Amplification products were quantified using the 2^−ΔΔCT^ method and were measured relative to level of GAPDH as the reference gene.

**Table 2:** The integrated optical density (IOD) and the positive expression areas of p-JNK, c-Jun, p-c-Jun, Sox-9 and COL2 measured by immunofluorescence

	NC		IL-1β		HBO		H+I	
	IOD	Positiveareas (μm^2^)	IOD	Positiveareas (μm^2^)	IOD	Positiveareas (μm^2^)	IOD	Positiveareas (μm^2^)
p-JNK	32.6±9.8	22.6±7.2	82.3±6.2*	70.4±8.8*	38.3±10.4^#^	25.6±12.7^#^	29.2±7.2^#^	20.4±9.2^#^
c-Jun	30.4±6.8	20.3±5.9	62.4±8.8*	52.6±9.8*	74.3±9.2^&^	69.2±10.2^&^	66.8±6.2^&^	60.8±8.2^&^
p-c-Jun	26.4±7.2	18.9±9.8	69.8±10.2*	58.4±12.7*	29.4±3.6^#^	22.4±5.8^#^	29.2±8.2^#^	20.5±8.4^#^
Sox-9	89.8±8.6	67.4±6.2	30.2±8.3*	29.3±9.5*	86.3±9.2^#^	70.4±9.8^#^	79.8±10.2^#^	66.4±12.2^#^
COL2	96.6±5.6	79.8±8.8	36.1±6.2*	28.2±6.2*	76.4±12.6^#^	72.3±10.2^#^	86.2±6.2^#^	79.6±7.4^#^

C, control; IL-1β, cells with 10ng/ml interleukin-1β; HBO, hyperbaric oxygen treated cells with 10ng/ml interleukin-1β; H+I, hyperbaric oxygen treated cells with 10ng/ml interleukin-1β and 40μM SP600125 (the JNK inhibitor). *P<0.05, significantly different from the control group; ^#^P<0.05, significantly different from the IL-1β group. ^&^P>0.05, no obviously difference from IL-1β group. Data are represented as the M±SEM of n=8. M, mean; SEM, standard error; n, sample size.

### Statistical analysis

All the data were expressed as means ± SEM. Experimental data were analyzed by one-way analysis of variance (ANOVA). Relative indices were analyzed by SPSS 13.0 software (SPSS, USA). Differences between two groups were measured with Student's test. A P value of less than 0.05 was considered as statistically significant.

## Abbreviations

HBO: hyperbaric oxygen
OA: osteoarthritis
IL-1β: interleukin-1β
TMJ: temporomandibular joint
ECM: extracellular matrix
TMJOA: temporomandibular joint osteoarthritis
COL2: type II collagen
JNK: c-Jun N-terminal kinase
p-JNK: phospho-JNK
c-Jun, p-c-Jun: phospho-c-Jun
Sox-9: SRY-box9
